# Environmental impacts of rare earth production

**DOI:** 10.1557/s43577-022-00286-6

**Published:** 2022-03-17

**Authors:** Petra Zapp, Andrea Schreiber, Josefine Marx, Wilhelm Kuckshinrichs

**Affiliations:** Institute of Energy and Climate Research-Systems Analysis and Technology Evaluation (IEK-STE), Forschungszentrum Jülich, Jülich, Germany

**Keywords:** Rare earth elements, Life cycle assessment (LCA), Environmental impacts, Ion-adsorption clays, Bastnäsite, Monazite, Eudialyte

## Abstract

Rare earth elements (REEs) are important raw materials for green technologies. However, REE mining and production uses techniques that are often not environmentally sustainable. Life cycle assessment (LCA) is a well-recognized method for evaluating the environmental impacts of products and technologies. This article provides an overview of the environmental impacts based on published LCA results of primary REE production. Existing major REE deposits (Bayan Obo in China, Mountain Pass in the United States, Mount Weld in Australia, ion-adsorption deposits in several Chinese southern provinces) and currently possible production routes are compared. Alternative minerals, such as eudialyte, are also discussed. The article shows which environmental effects can be minimized by technology optimization and environmental safety strategies. Additionally, some of the environmental impacts discussed, may be difficult to mitigate, as they depend on the mineral type. Activities along the complex process chain of REEs production that have particularly high environmental impacts are identified.

**Graphical abstract:**

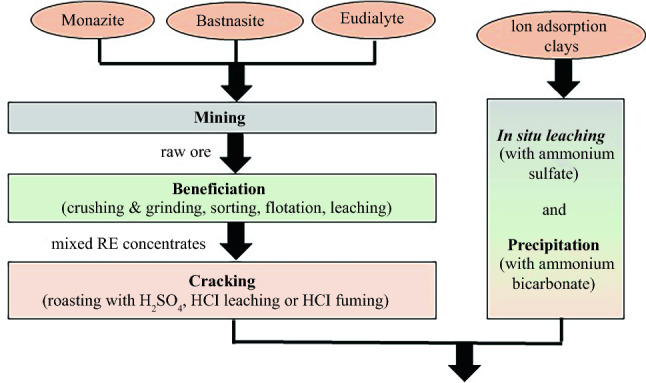

## Introduction

Ever since the increasing importance of rare earth elements (REEs) for green technologies, such as wind turbines, electric vehicles, and low-energy lighting, there has been a parallel discussion concerning environmental impacts caused by the mining and production of REEs. The growing interest was disclosed in a recent review of life cycle assessment (LCA) studies,^1^ which have their focus on environmental impacts of REE production. In the last five years, at least 27 studies were conducted evaluating environmental impacts of various REE products and supply chains. For example, Schulze et al.^2^ and Deng and Kendall^3^ considered REE production from ion-adsorption clays (IACs), Marx et al.^4^ and Arshi et al.^5^ compared REE production from various mineral types, Vahidi and Zhao analyzed in particular the separation of REEs through solvent extraction^6^ and the production of RE metals via molten salt electrolysis,^7^ and Schreiber et al.^1^ published a comprehensive review of LCA activities involving primary REE production. Though detailed industry data are normally not available, LCA studies provide sufficient insight in the cause-and-effect chains to allow a basic evaluation of various primary REE supply routes.

Like all material processing, the REE supply chain is also associated with environmental impacts. They are mainly related to the geology of a deposit, mineral type and composition, the methods of extraction, local supply of energy and auxiliary materials, and regulatory conditions that mitigate environmental impacts. Hence, the environmental impacts vary considerably. In the course of the “Legislative Report on Human Rights and Environmental Due Diligence of Businesses” (adopted by the European Parliament in March 2021),^8^ recommending that the EU Commission introduce an EU-wide supply chain law, the discussion of environmental impacts will gain further momentum. For example, in June 2021, the German Bundestag had already passed a law on corporate due diligence in supply chains.^9^ The law aims to better protect human rights and the environment in the global economy. These include the prohibition of child labor, protection against slavery and forced labor, adequate wages, the right to form trade unions, and access to food and water. The law also considers risks to the environment, such as the release of hazardous substances or polluted water.

China represents the largest REEs producer worldwide with 140,000 t rare earth oxide (REO) equivalents in 2020 (58%)^10^ and Bayan Obo is its largest deposit. The ore from Bayan Obo is processed in Baotou, 150 km away. REOs are also produced in Sichuan and from IACs in several southern provinces. It is difficult to find precise production data because illegal mining produces a significant amount of REOs, which is especially true for IACs.^11^ Also, for legal mining reliable public data are scarce. Since 2015, there have been serious attempts to consolidate China’s RE industry into six large corporations controlled by the central government or by provincial and municipal governments to establish industrial order, address environmental issues, and internalize pollution costs.^12^

Following a temporary shutdown of the Mountain Pass production site in 2016 and 2017, the United States is again the world’s second-largest producer with 38,000 t REO in 2020 (16%).^10^ Currently, RE production only takes place up to RE concentrates at Mountain Pass. The final processing occurs in Asia.

Australia is currently the fourth-largest producer with 17,000 t REO in 2020 (7%) after Myanmar with 30,000 t REO (12.5%).^10^ The Australian production decreased significantly in 2020 compared with 2018 and 2019, both with 21,000 t of REO,^10^ due to the COVID-19 pandemic. Australian’s mining company Lynas Corporation operates the Mt Weld mine in Western Australia and a processing plant in Gebeng, Malaysia (near the city of Kuantan), where the RE concentrate from Mt Weld is further processed to individual REOs.

Although some European deposits are already identified,^13^ for example, in Kvanefield (Greenland) and Norra Kärr (Sweden),^14^ only very limited mining activities have occurred thus far. Other potential mining sites outside Europe include Thor, Hoidas, and Strange Lake (Canada), Bear Lodge (United States), Nolans Bore (Australia), and Steenkampskraal (South Africa),^15^ of which only the last two projects have achieved “permitted status” to date.^16^

Not only mining and processing of REOs are concentrated in China but also most of the RE smelting industry (95%). Some smaller capacities exist in Vietnam and Laos (5%).^17^ Smelting capacity in Japan and other Western countries is negligible.

Various deposits present different mineral types. While the Bayan Obo ore is a mixture of bastnäsite (REFCO_3_) and monazite (REPO_4_), bastnäsite prevails in Sichuan and Mountain Pass, monazite in Mt Weld, and eudialyte (N_15_ [M(1)]_6_ [M(2)]_3_ [M(3)] [M(4)] Z_3_ [Si_24_O_72_] O’_4_X_2_ with N=Na, Ca, K, Sr, REE, Ba, Mn, H_3_O^+^; M(1)=Ca, Mn, REE, Na, Sr, Fe; M(2) = Fe, Mn, Na, Zr, Ta, Ti, K, Ba, H_3_O^+^; M (3, 4) = Si, Nb, Ti, W, Na; Z = Zr, Ti, Nb; Oʹ = O, OH^−^, H_2_O; X = H_2_O, Cl^–^, F^–^, OH^–^, CO_3_^2–^, SO_4_^2–^, SiO_4_^4–^)^18^ in Norra Kärr. IACs are in the Chinese southern provinces (CSP) and in Myanmar. In IACs, REOs are adsorbed on the surface of alumino-silicate minerals (e.g., kaolinite [Al_2_Si_2_O_5_(OH)_4_] * REE). The mineral types affect the level and type of environmental pollution as well as the applied technology.

## Environmental impacts

The following brief analysis presents the key environmental impacts that may occur along the process chain from mining of various REE minerals to REE metal refining. The LCA method assigns emissions to so-called impact categories that address not only the greenhouse gas effect, but also other environmental impacts such as acidification, eutrophication, and toxicities. Based on literature or modeled process data, process chains are compared representing different mineral types and corresponding processing technologies, but also different technology standards. These process chains do not represent real supply chains of individual companies but are to be understood as generic REE production pathways. A distinction is made between environmental impacts that can directly be minimized by, for example, process adjustments or cleaning techniques for exhaust gas and wastewater, and those where direct mitigation options do not exist, such as those caused by mineral composition.

## Description of general process chains

**Figure 1:**
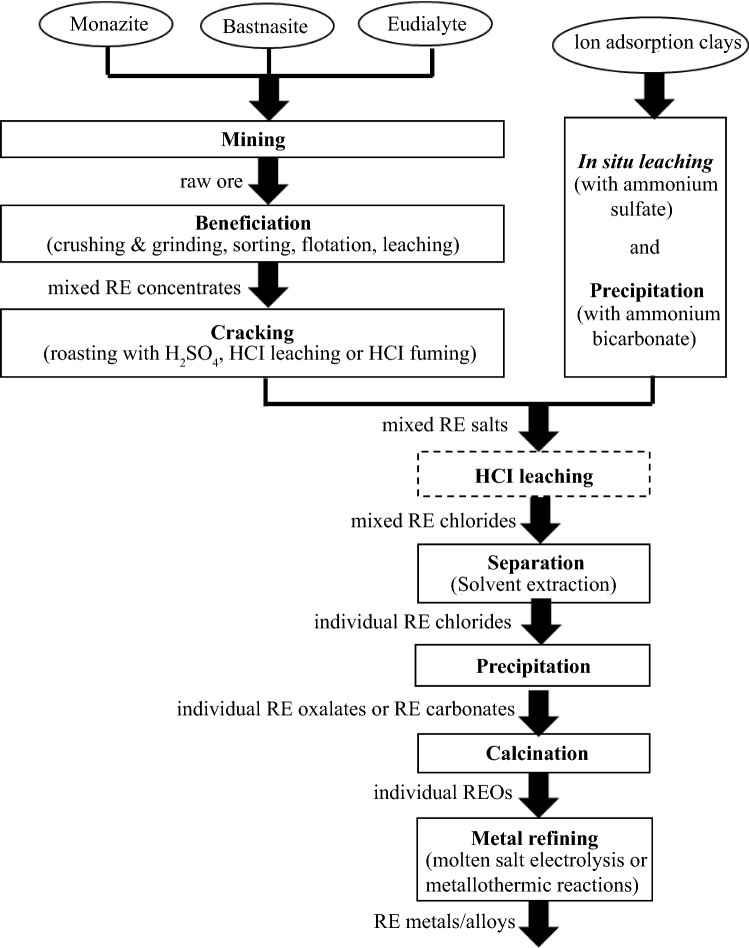
Simplified process chain of RE production (dashed line means optional process).

The general process chains for bastnäsite, monazite, bastnäsite/monazite mixed ore, and eudialyte are similar, even if they start from different minerals (**Figure ****1**).^4,19^ The mined ore is crushed and then wet ground. In the subsequent flotation process, the ore is separated from accompanying minerals. Beneficiation efficiency and REO concentration in the concentrates vary depending on the flotation process. A cracking process in which the concentrates are converted into soluble RE salts (sulfates, chlorides, carbonates) follows. In Baotou and Kuantan cracking is done by roasting with sulfuric acid (H_2_SO_4_). In Sichuan, the pure bastnäsite is cracked via hydrochloric acid leaching. Eudialyte (Norra Kärr) is cracked by a dry digestion with hydrochloric acid (HCl) (called “fuming”).^20^ The former Molycorp Inc cracking process (at Mountain Pass) was subject to confidentiality, so no technical details were provided. It is assumed that HCl was used for cracking as chlor-alkali electrolysis was available at the site. The REE salts are then separated from remaining gangue and secondary elements by different leaching and precipitation processes. Solvent extraction to separate the REEs follows the same procedure for all process chains. The separated REE chlorides are precipitated as carbonates or oxalates. These are calcined to REO. Reduction to the metal is carried out by molten salt electrolysis (Nd, Pr, La, Ce), calciothermic (Dy), and metallothermic reduction (Sm).^7^

In the case of the IACs, the extraction of the REEs takes a different route.^21,22^ Neither mining, crushing, grinding nor cracking is performed but an *in situ* leaching process with ammonium sulfate. Processing of the leached REE sulfates is carried out as previously described.

Pure bastnäsite is easier to beneficiate than mixed ores or pure monazite and, therefore, generates less environmental impacts.^23^ In eudialyte deposits of high REE concentrations up to 10% have been found so far.^24^ Other huge advantages of eudialyte compared to other minerals is the higher proportion of heavy REEs (up to 50% of total REO) and the very low concentration of ThO_2_ and U_3_O_8_ with 26 ppm and 18 ppm, respectively, containing the radioactive elements ^232^Th and ^238^U.^14,19,24^ The activity (decay rate) is 3566 Bq ^232^Th/g ThO_2_ and 10,470 Bq ^238^U/g U_3_O_8_.^25^ Monazite contains up to 200,000 ppm ThO_2_ and up to 160,000 ppm U_3_O_8._^26^ However, for the monazite deposit of Mt Weld lower values are given (750 ppm ThO_2_, 30 ppm U_3_O_8_).^25^ For the mixed bastnäsite/monazite deposit at Bayan Obo, the ThO_2_ concentration is reported to be 320 ppm,^27^ 400 ppm,^26^ and 700 ppm^25^ and the U_3_O_8_ concentration is given as 2 ppm.^25^ For the bastnäsite deposit in Sichuan, 400 ppm ThO_2_ and 40 ppm U_3_O_8_ are reported; for Mountain Pass 200–10,000 ppm ThO_2_ and 20 ppm U_3_O_8._^25^ The radioactivity of IACs is generally low^28^ and is reported to be 50 ppm each for ThO_2_ and U_3_O_8._^25^ Other environmental impacts are particularly high when REEs are extracted from IACs, as much wastewater is produced with high concentrations of ammonium sulfate (> 150 mg/l ammonium, > 5000 mg/l sulfate^26^) and heavy metals.^16^ In general, a lot of tailings, which are mixtures of crushed rocks and processing fluids from mills and concentrators, and waste are produced from REE processing that is partially radioactive and highly hazardous. The activity of the leaching sludge and acidic process slag is much higher than that of the raw ores.^25,27,29^ The waste contains the acids and organic solvents.^16^ New techniques such as bioleaching and molecular recognition technology instead of solvent extraction could save a lot of energy and waste.^16^ However, these techniques exist so far only in the laboratory, and it will take years to reach technical maturity at industrial production scales.

## Mining

Radioactive dust caused by blasting and mine sewage contribute mainly to the environmental impact categories: particulate matter formation (PM) and ionizing radiation (IR). The quantities of blasting agent depend on the stripping rate. The more rock that has to be blasted and removed, the more dust is generated. The amount of dust can be reduced by irrigation systems, as provided in Mountain Pass and Norra Kärr. Mine sewage contains heavy metals that are released into the soil when discharged uncontrolled, such as in China.^26,30,31^ The energy demand cannot be influenced very much because drilling expenses for blasting and transport within the mine depend on the specific site conditions.

## Beneficiation

Crushing, grinding, and (magnetic) sorting require energy (electricity, diesel) that depends on the hardness of the rock, which is highest for the Bayan Obo deposit, followed by the Mountain Pass, Mt Weld, and Norra Kärr deposits.^4^ The environmental impacts of flotation result from energy requirements and from the production of flotation chemicals. However, they do not play a major role in assessing the environmental impacts of the overall chain, as they are only added in small quantities (approximately 3 kg/t ore). Flotation tailings (up to 40 t/t REO depending on the flotation process) are discharged into open tailings ponds. Depending on the site-specific construction features of the tailing ponds (e.g., sealing), heavy metals and inorganic phosphorus-containing compounds may seep into the soil. The leakage rate is high for Baotou deposit because the tailings pond has no liner system or vegetation cover. Because the dam is located about 35 m above the Yellow River, the tailings pond poses increasing toxicity risks to water, soil, and air through leakage, dust formation, and rain erosion.^26^ The Baotou tailings are contaminated with radioactive thorium with a mean concentration of 5% and high concentrations of dissolved solids, chlorides, sulfates, fluorides, ammonium, boron, manganese, and iron.^26^ Western sites such as Mountain Pass and Mt Weld are equipped with liner systems and can respond to leakage.^14,32^ However, even at these facilities, the tailings ponds have a high potential for environmental damage should accidental releases occur.

## Cracking

The two main cracking processes are roasting with H_2_SO_4_ (as used in Baotou and Kuantan) and different reactions with HCl (as used for bastnäsite in Sichuan and for eudialyte) (Figure 1). The consumption of H_2_SO_4_ for acid roasting is determined by the chemical reaction of REFCO_3_ in bastnäsite and of REPO_4_ in monazite to RE sulfate according to reactions (1) and (2), respectively.^23^ Depending on the technical standard and environmental protection measures, the quantities of H_2_SO_4_ vary between modern large-scale plants and small plants with low environmental standards.

1 $$ {\text{bastn}}\"a {\text{site: }} {\text{2 REFCO}}_{{3}} + {\text{3 H}}_{{2}} {\text{SO}}_{{4}} \to {\text{RE}}_{{2}} \left( {{\text{SO}}_{{4}} } \right)_{{3}} + {\text{2 HF}} + {\text{2 H}}_{{2}} {\text{CO}}_{{3}} $$

2 $$ {\text{monazite}}: {\text{2 REPO}}_{{4}} + {\text{3 H}}_{{2}} {\text{SO}}_{{4}} \to {\text{RE}}_{{2}} \left( {{\text{SO}}_{{4}} } \right)_{{3}} + {\text{2 H}}_{{3}} {\text{PO}}_{{4}} $$

Bastnäsite produces harmful hydrogen fluoride (HF) emissions during acid roasting, which are determined by the mineral composition and therefore cannot be influenced. However, they can be reduced by up to 99% through exhaust gas scrubbing. Sulfur dioxide emissions resulting from excess H_2_SO_4_ can also be significantly reduced by exhaust gas scrubbing.

Reaction equations (3–6) show the cracking of pure bastnäsite with HCl.^23^ The formation of large amounts of HF is avoided.

3 $$ 3\,{\text{REFCO}}_{3} + {\text{heat}} \to {\text{RE}}_{2} {\text{O}}_{3} + {\text{REF}}_{3} + 3\,{\text{CO}}_{2} $$

4 $$ {\text{RE}}_{{2}} {\text{O}}_{{3}} + {\text{6 HCl}} \to {\text{2 RECl}}_{{3}} + {\text{3 H}}_{{2}} {\text{O}} $$

5 $$ {\text{REF}}_{{3}} + {\text{3 NaOH}} \to {\text{RE}}\left( {{\text{OH}}} \right)_{{3}} + {\text{3 NaF}} $$

6 $$ {\text{RE}}\left( {{\text{OH}}} \right)_{{3}} + {\text{3 HCl}} \to {\text{RECl}}_{{3}} + {\text{3 H}}_{{2}} {\text{O}} $$

Due to confidentiality by Molycorp Inc, no reaction equation can be given for the cracking process of bastnäsite in Mountain Pass.

The so-called “fuming” process with HCl transforms eudialyte into a mixture of metal salts and a siliceous secondary precipitate.^20^

It is assumed that exhaust gas scrubbing is only installed in modern Chinese large-scale plants. However, because there are also medium-sized and small plants in China with minimal to no exhaust gas scrubbing, a significant release of HF because of acid roasting and other emissions can be expected. The capacities of the different plant types are not exactly known, because no plant-specific Chinese data are publicly available. It is assumed that exhaust gas scrubbing is installed in all western plants but only in modern Chinese large-sized plants.

## Leaching

After cracking, further gangue and accompanying elements are separated from the aqueous sulfate solutions. The leaching and precipitation processes require large amounts of chemicals (e.g., HCl, H_2_SO_4_, NaOH), and their production is associated with considerable environmental pollution that clearly contributes to the environmental impacts of the process chain. They can be reduced by recycling of chemicals (e.g., HCl), as was done at Mountain Pass. The resulting leaching sludge has varying levels of radioactivity depending on the ore. The limit for classification as radioactive waste (1 Bq/g for ^232^Th) is often significantly exceeded by the leaching sludge. As already mentioned for the flotation tailings, the environmental impacts of leaching sludge depend on the type of storage. When operated in accordance with regulations, leakage occurs through infiltration. Liner systems can reduce the environmental impacts.

## Solvent extraction, precipitation, and calcination

The separation of the individual REEs is very complex, due to their similar physical and chemical properties. So far, solvent extraction is the most common method for industrial production. Up to hundreds of mixer and settler stages may be assembled to separate all the individual REEs. This process leads to a high demand of chemicals, especially HCl. Compared to inorganic chemical consumption, only small amounts of organic chemicals such as the extractants P204 (2-ethylhexyl phosphoric acid mono 2-ethylhexyl ester) and N235 (trialkyl amine R_3_N, R = C8–C10) are consumed.^6,23^ The requirement of chemicals is given by the share of each REE in the ore. However, demand can be significantly reduced, for example, by HCl recycling^4^ and reprocessing of wastewater.

The subsequent precipitation of the separated RE chlorides to RE oxalates and RE carbonates is carried out with oxalic acid and ammonium bicarbonate, respectively. The environmental impacts occur during production of the precipitation chemicals and not during the precipitation process.

The environmental impacts of calcination of RE oxalates and RE carbonates to REOs are only marginal. Choosing an environmentally friendly energy source for the tunnel kiln could further reduce CO_2_ emissions and fossil fuel consumption.

## Metal refining

Besides REO, the main inputs into the molten salt electrolysis are electricity, electrolytes (RE fluoride (REF_3_), lithium fluoride (LiF)), graphite (anode), and tungsten or molybdenum (cathode). Production of electrolytes, electricity consumption, and used cathodes contribute most to the impacts. The amount of REF_3_ and LiF could be reduced considerably by dust filter and recycling.^4,33^ In contrast, the electricity consumption of approx. 8–12 kWh/kg RE metal (current efficiency is 75–80%)^7^ cannot be reduced significantly because a voltage reduction would lead to a lower energy input into the electrolysis cell and thus to a disturbance of the heat balance. The size of the metal reduction plant plays a central role for the environmental impact. It is decisive whether it is a small backyard plant of 3–4 kA or a large state-owned plant of 30 kA equipped with an exhaust gas scrubbing.^33^ Lower environmental impacts can be achieved by decarbonized electricity generation, automated process control, exhaust gas cleaning, and recycling of used electrolytes.^4^ This effect was shown in studies that compared various Chinese electrolysis scenarios.^4,17,33^

## Ion-adsorption clays

In contrast to the extraction of REEs via open-pit mining from bastnäsite and monazite, the REEs adsorbed in IACs are leached via *in situ* leaching according to reaction shown in Eq. (7).^34^

7 $$ \left[ {{\text{Al}}_{{2}} {\text{Si}}_{{2}} {\text{O}}_{{5}} \left( {{\text{OH}}} \right)_{{4}} } \right]_{{\text{a}}} *{\text{ b RE}}^{{{3} + }} {\text{ + 3 b NH}}_{{4}}^{ + } \to \left[ {{\text{Al}}_{{2}} {\text{Si}}_{{2}} {\text{O}}_{{5}} \left( {{\text{OH}}} \right)_{{4}} } \right]_{{\text{a}}} *\left( {{\text{NH}}_{{4}}^{ + } } \right)_{{{\text{3b}}}} + {\text{b RE}}^{{{3} + }} $$

Unlike processing bastnäsite, monazite, and eudialyte no beneficiation is required for IACs but the REEs are extracted solely via hydrometallurgical processes. *In situ* leaching is followed by precipitation with ammonium bicarbonate before solvent extraction (Figure 1).

Large quantities of ammonium sulfate are used as leaching agent during *in situ* leaching of IACs. Public data are only partially available, especially since a large share of IACs are mined illegally. The environmental impacts depend on whether the ammonium sulfate solution remains in the soil or is pumped off and treated or recycled. If it is recycled, emissions can be reduced significantly, especially for the environmental category of eutrophication (EP). The amounts of ammonium sulfate reported in the LCA studies vary substantially from 4 kg^3^ to 80 kg^21^ per kg REO due to uncertainty in publicly available operating data. The impact category marine eutrophication (EP_Marine_) is mainly affected by ammonium emissions into soil and water, while the increase of other impact categories (e.g., ecotoxicity potential freshwater (ETP_Freshwater_), eutrophication potential freshwater (EP_Freshwater_), human toxicity potential (HTP), particulate matter formation (PM), global warming potential (GWP)) is influenced by the production of ammonium sulfate.^21^ The high ammonium sulfate concentration of 3500–4000 mg/l found in groundwater^35^ suggests a high consumption of ammonium sulfate for in situ leaching. On the other hand, this suggests that the leaching agent remains in the soil and subsequently pollutes the groundwater.

## Results of LCA comparison

So far, no emissions based on measured data from the facilities have been published in the LCA studies. Rather, general thresholds prescribed by the Chinese Ministry of Ecology and Environment (formerly MEP) or Environmental Protection Agency (EPA) are given in some studies, which neither reflect the specific ore compositions nor the amount of chemicals used.^36,37^ There is also a lack of data on the real environmental impacts of mine wastewater as well as effluents from flotation, exhaust gas scrubbing, leaching, and wastewater neutralization. In addition, radioactive emissions, especially caused by ^232^Th, are not considered in most studies.^1^ Some of the emissions could be reduced by preventing leakage of pollutants (e.g., heavy metals, inorganic salts, toxic organic chemicals) into water and soil through appropriate sealing of tailing ponds.^14,32^

**Figure 2:**
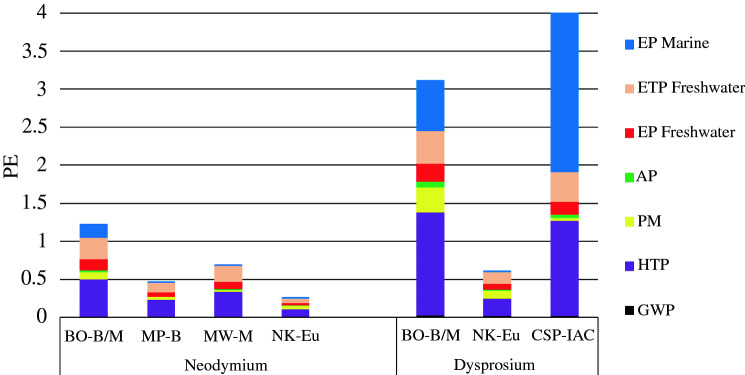
Normalized impacts of 1 kg of neodymium and dysprosium. *BO* Bayan Obo/Baotou, *MP* Mountain Pass, *MW* Mt Weld/Kuantan, *NK* Norra Kärr, *CSP* Chinese southern provinces, *B/M* bastnäsite/monazite, *B* bastnäsite, *M* monazite, *Eu* eudialyte, *IAC* ion-adsorption clay.

**Figure ****2** presents the sum of seven normalized impact categories of neodymium and dysprosium production for the generic process chains of different mining sites as they were published in three studies.^4,19,21^ Normalized figures allow the comparison and adding of different environmental impacts by setting each impact in relation to the same impact induced by an average person per year (normalization factors). The result of the normalization step (relative approach) is expressed in person equivalents (PE).

**Figure 3:**
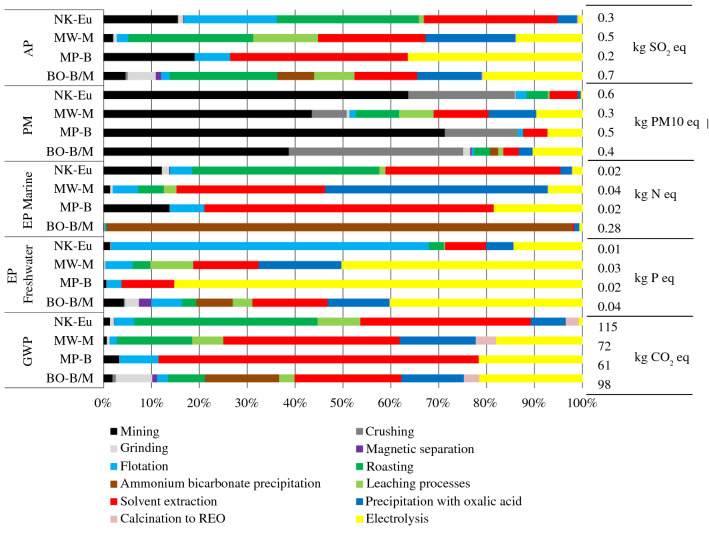
Relative contribution (%) of process steps (marked in the legend) to five normalized impacts (GWP, EP_Freshwater_, EP_Marine_, PM, AP) of 1 kg neodymium; the right axis additionally shows the absolute figures of the environmental impacts of each of the four process chains (based on eudialyte at Norra Kärr, monazite at Mt Weld, bastnäsite at Mountain Pass, and the mixed bastnäsite/monazite ore at Bayan Obo) in kg equivalents.

**Figure 4:**
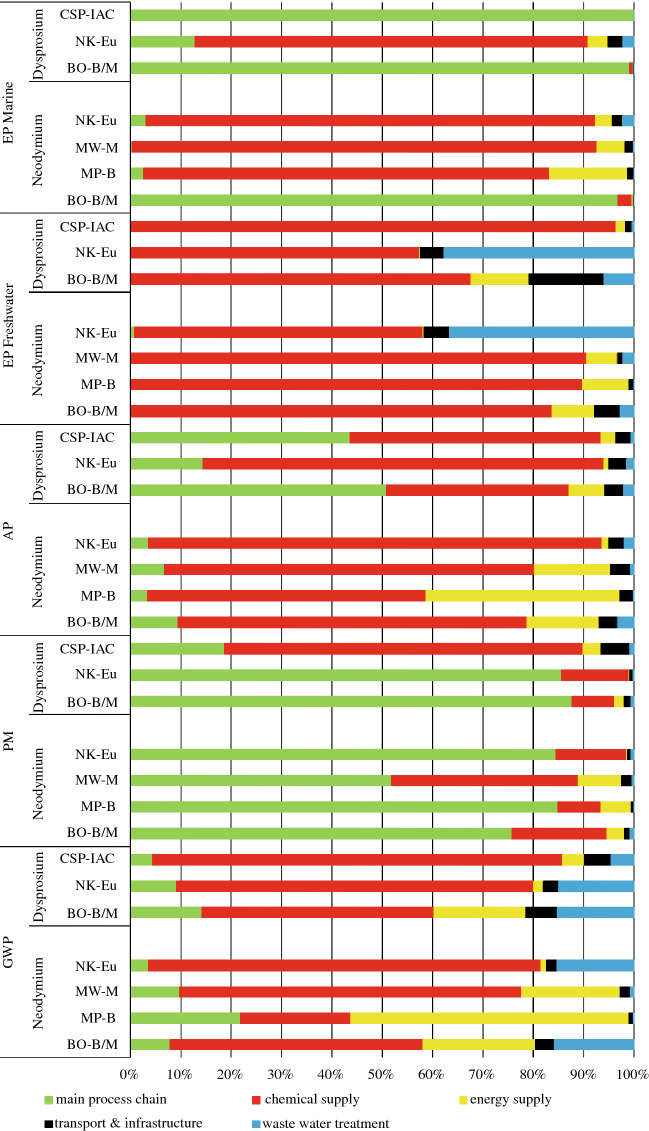
Contribution of processes of the main RE process chain as well as upstream and downstream processes to the entire process chain.

In general, the production of dysprosium has a higher environmental impact than that of neodymium because dysprosium is always present in lower concentrations in the ores. Eudialyte has the highest dysprosium content (0.03%) in comparison to the Bayan Obo ore and the IACs, whose dysprosium content is about 0.0037 percent. The dysprosium production based on IACs induces the highest environmental impacts of all process chains (Figure 2). Especially ammonium emissions during *in situ* leaching with ammonium sulfate cause the very high impact of EP_Marine._^21^ Neodymium production from Norra Kärr eudialyte shows the lowest environmental impact,^19^ followed by bastnäsite from Mountain Pass, monazite from Mt Weld/Kuantan and bastnäsite/monazite from Bayan Obo/Baotou.^4^ However, it must be considered that the eudialyte REE chain is based only on laboratory data. The case of REE production from Mountain Pass bastnäsite shows the effectiveness of measures, such as recycling of saline wastewater to save chemicals, cleaner power generation using a natural gas-fired cogeneration plant, and an alternative cracking process without roasting, which was used by the former operator Molycorp Inc. Still, these measures do not reduce all environmental impacts. For example, PM is determined by the geologic properties of the deposit and can only be reduced to a small extent by an irrigation system. Changes in processing procedure are also rather unlikely, as they are determined by the mineral type. In contrast, improvements in process efficiency have an impact. Also, exhaust gas treatment of electrolyzers and modern sludge treatment concepts can add up to a recognizable improvement as assumed for the Mt Weld/Kuantan process chain.^4^

Mining and crushing are the largest contributors to PM (approximately 50–90%; Figure 3). Production of ammonium bicarbonate for the precipitation step to RE carbonates in case of the Bayan Obo/Baotou process chain dominates the impact category EP_Marine_ (> 98%). Flotation and magnetic sorting have lower impacts due to their lower energy and chemical requirements as compared with cracking and hydrometallurgical processes. Roasting, solvent extraction, precipitation to REE oxalates and calcination contribute most to GWP and acidification potential (AP) mainly caused by electricity demand required for chemical supply. Roasting also causes AP due to direct process emissions of HF and sulfur dioxide. The share of the precipitation chemicals on the total environmental impacts amounts to 1–20% for the Bayan Obo/Baotou production pathway and even 10–40% for Mt Weld/Kuantan pathway, depending on the individual impact category. Molten salt electrolysis causes impacts of EP, AP, HTP, ETP_Freshwater_, PM, and GWP.^4,7^ GWP is caused by CO_2_, CF_4_, and C_2_F_6_; HTP, AP and ETP_Freshwater_ by HF and PM by dust particles. Phosphate emissions during cathode production causes freshwater eutrophication potential (EP_Freshwater_) and HF emissions during REF_3_ production account for HTP and AP.

The breakdown of process chain components into upstream and downstream processes (e.g., chemical and energy supply, transport processes, wastewater treatment) clearly shows the dominant share of chemicals in almost all impact categories (Figure 4). PM is dominated by dust emissions during mining of bastnäsite, monazite, and eudialyte. Ammonium emissions during *in situ* leaching of IACs and REE carbonate precipitation with ammonium bicarbonate (Baotou) are the main contributor to EP_Marine_. Energy consumption is mostly reflected in GWP and AP. The share of transport and infrastructure facilities on the total environmental impacts is negligible (Figure 4).

## Conclusion

Many previous LCA studies provide useful insights into REE production and a good understanding of its environmental consequences. Because the majority of REEs are produced in China, data availability poses a major challenge. The LCA studies showed that each REE mineral and deposit is different and requires an individual analysis of the entire processing route. Therefore, despite many efforts, there is still no complete picture of the environmental impacts associated with REE production. In particular, the environmental analysis of illegal REE production, mainly IACs, is still a blind spot due to the lack of data. Here, scenarios and sensitivity analyses are often used. Moreover, especially for metal refining, it is unclear how much of the RE metals/alloys are produced and with what efficiency (large state-owned refineries with exhaust gas cleaning or small, mostly private backyard operation sites without exhaust gas cleaning). However, the LCAs have highlighted the main problems, such as large quantities of chemicals needed to process REEs and the large quantities of tailings generated during beneficiation, extraction and separation that contain the naturally occurring radionuclides ^232^Th and ^238^U and their decay products. These radioactive elements can enter the environment through air, wastewater and rain leaching. The entire process chain has potential for improvement through emission treatment technologies and also recycling of chemicals (e.g., HCl) generated during processing. Closing illegal mines and raising environmental standards should be addressed as a high priority to reduce environmental impacts. Reinforcing responsibilities along cross-border process chains, the supply chain laws mentioned in the introduction could probably be helpful in this regard. Supplying methodically stringent data, LCAs are a credible means to promote this development.

In addition, new primary resources should be identified that have high REE content, as few radioactive associated elements as possible and are located outside sensitive ecosystems as alternative REE production pathways to China. Moreover, research into new or improved processing technologies and reprocessing of industrial waste streams should be pursued. Other, secondary REE resources and increased end-of-life recycling of REE-containing consumer products are additional options. Despite extensive research, mostly on a laboratory scale, only about 1% of REEs are actually recycled.^38^ The reasons for this are inefficient collection and technological problems. The complex chemical separation of REEs is a big challenge and a chief barrier to widespread recycling activities. In 2018, Apple rolled out a Robot for iPhone dismantling. Processing 100,000 iPhones has the potential to recover 11 kg of REE.^39^ Dramatically improving the recycling of REEs is an absolute necessity. LCAs are needed to evaluate improvement options.
